# USP10 suppresses ABCG2-induced malignant characteristics of doxorubicin-resistant thyroid cancer by inhibiting PI3K/AKT pathway

**DOI:** 10.1007/s10863-023-09986-3

**Published:** 2023-11-03

**Authors:** Jianwei Sun, Qian Xiang, Ding Ding, Nan Yan

**Affiliations:** 1 Department of Ultrasound, Fifth Affiliated Hospital of Kunming Medical University, 17 South Goldenlake Road, Gejiu, 661000 China; 2 Department of Endocrinology, Fifth Affiliated Hospital of Kunming Medical University, 17 South Goldenlake Road, Gejiu, 661000 China

**Keywords:** Thyroid cancer, USP10, ABCG2, Doxorubicin resistance, Pathways

## Abstract

Doxorubicin (DOX) is the most extensively used drug in the chemotherapy of thyroid cancer (TC). However, the existence of DOX resistance is not conducive to TC treatment. Here, we investigated the role of USP10 in DOX-resistant TC and explored the underlying molecular mechanism. CCK-8 assay was used to measure cell viability in thyroid cancer FTC133 and DOX-resistant FTC133-DOX cells. RT-qPCR and western blot were used to evaluate USP10 expression. Cell migration, invasion, and apoptotic assays were conducted. Western blot was used to detect cellular signaling proteins, EMT-related proteins, and apoptosis-related proteins. We found a lower expression of USP10 in the human TC cell line FTC133 as compared to the normal human thyroid Htori-3 cells. Notably, USP10 expression was further reduced in DOX-resistant (FTC133-DOX) cells compared to the FTC133 cells. FTC133-DOX cells had increased invasion, migration, and EMT properties while less apoptosis by activating the PI3K/AKT pathway. Interestingly, overexpressing USP10 increased the chemosensitivity of FTC133 cells to DOX therapy. Overexpressing USP10 inhibited invasion, migration, and EMT properties of FTC133-DOX cells and promoted apoptosis. Mechanistically, overexpressing USP10 inhibited PI3K/AKT pathway by activating PTEN. Furthermore, overexpressed USP10 controlled all these processes by downregulating ABCG2. This study demonstrates that USP10 could reduce DOX-induced resistance of TC cells to DOX therapy and could suppress TC malignant behavior by inhibiting the PI3K/AKT pathway. Furthermore, USP10 targeted ABCG2 to inhibit all these malignant processes, therefore, either increasing USP10 expression or inhibiting ABCG2 could be used as novel targets for treating DOX-resistant thyroid cancer.

**Supplementary Information:**

The online version contains supplementary material available at 10.1007/s10863-023-09986-3.

## Introduction

Thyroid cancer (TC) is the most common endocrine malignancy, and its incidence has increased significantly over the past three decades(Seib and Sosa 2019). Despite advancements in medicine and the availability of new and more promising therapeutics(Laha et al. 2020), doxorubicin (DOX) remains the most extensively used drug in the chemotherapy of thyroid cancer. DOX is an antibiotic derived from the Streptomyces peucetius bacterium(Robey et al. 2018). Unfortunately, resistance to DOX therapy is common which is not favorable to thyroid cancer treatment (Xiong et al. 2021). Therefore, it is the need of time to investigate the molecular mechanism by which DOX induces resistance in thyroid cancer.

One study has found that resistance to DOX therapy fails to eradicate anaplastic thyroid cancer or even to stop tumor progress was closely related to adenosine triphosphate-binding cassette subfamily G member 2 (ABCG2) (Zheng et al. 2010). In order to reverse ABCG2-induced drug resistance, extensive research has been conducted to explore the signaling pathways that are related to ABCG2-induced drug resistance (Po et al. 2020; Singh et al. 2011; Zhang et al. 2019). Research suggests that the phosphatidylinositol 3-kinase/protein kinase B (PI3K/AKT) pathway is involved in regulating the expression of ABCG2 (Liu et al. 2021) and inhibiting the PI3K/AKT pathway using LY294002, or rapamycin counteracted the protective effects of ABCG2 against chemotherapeutic drug treatment (Wang et al. 2019). PI3K/AKT pathway has been regarded as a key link that modulates the multidrug resistance of cancers (Liu et al. 2020). In one study, Liu et al., revealed that the PI3K/AKT pathway is involved in cell proliferation and tumor growth in thyroid cancer (Liu et al. 2018). Similarly, Peng et al., also found that the PI3K/AKT pathway is related to drug resistance of rapamycin in thyroid cancer (Bian et al. 2020). In addition, it has been shown that modulating PI3K/AKT signaling pathway could successfully reverse DOX resistance in breast neoplasm cells (Chen et al. 2018). Therefore, inhibiting PI3K/AKT pathway might have a potential therapeutic effect against doxorubicin resistance in thyroid cancer. Phosphatase and tensin homologue (PTEN) as a prime antagonist of PI3K is the negative regulator of PI3K/AKT pathway (Haddadi et al. 2018). A study found that up-regulation of PTEN reversed P-glycoprotein-mediated drug resistance in cancer cells (Shi et al. 2020). Thus, regulating PTEN using some appropriate modulators could be used as a novel approach to inhibit PI3K/AKT mediated ABCG2 overexpression in drug-resistant cancer cells.

Ubiquitin-specific peptidase 10 (USP10) is an important member of the ubiquitin-specific peptidases (USPs) family, which has enormous significance in diverse cellular processes and many human diseases (Xiong et al. 2020). Sun et al. 2018 confirmed that USP10 inhibits lung cancer cell growth and invasion by upregulating PTEN (Sun et al. 2018). He et al., also illustrated that USP10 inhibits non-small cell lung cancer cell proliferation by restoring PTEN activity (He et al. 2021). Studies have shown that abnormal USP10 expression is involved in epithelial-mesenchymal transition, cell migration, cancer, and immunity in thyroid tissue (Magne et al. 2016). However, it is not known by which mechanism USP10 mediates these processes, especially in the case of thyroid cancer. In order to provide a molecular target for early diagnosis and treatment of thyroid cancer, here, we examined the role of USP10 against ABCG2-mediated malignant biological behavior of DOX-resistant thyroid cancer cells and shed light on the molecular mechanism by which USP10 mediated these processes.

## Materials and methods

### Cell culture

The human normal thyroid cell line Htori-3 (#XY688) and human thyroid cancer cell lines FTC133 (#XY317) and TPC-1 were purchased from Shanghai Jiya Biotechnology Co., Ltd (China). Cells were cultured in Dulbecco’s modified Eagle’s medium (DMEM)/F-12 (Pierce, USA) supplemented with 10% fetal bovine serum (FBS), and 100 µg/ml streptomycin and 100 IU/ml penicillin at 37 °C with 5% CO_2_ conditions.

### Construction of drug-resistant cell line and treatment

FTC133 and TPC-1 cells were incubated with increasing concentrations of DOX (from 10 to 400 nmol/L) to construct DOX-resistant FTC133 cells (FTC133-DOX) and TPC-1 cells (TPC-1-DOX) [39–42]. Briefly, FTC133 and TPC-1 cells were incubated with 10 nmol/L DOX and the drug concentration was doubled each time the treated cells reached the growth rate of the untreated cells, until the final concentration of 400 nmol/L DOX was applied. FTC133 and TPC-1 cells which gradually adapted to the higher DOX concentration means that FTC133 and TPC-1 cells developed into FTC133-DOX and TPC-1-DOX.

In order to overexpress USP10 and ABCG2 in FTC133 cells according to experimental needs, human USP10 or ABCG2 was amplified from the HepG2 cDNA library and subsequently subcloned into the pCDNA3.0 plasmid. And then transfected pcDNA-USP10 or pcDNA-ABCG2 into FTC133 cells using Lipofectamine 2000 (Invitrogen, USA) according to the manufacturer’s instructions.

### Cell viability assay

To find the half maximal inhibitory concentration (IC50) of DOX, cell viability assay was performed using a CCK-8 kit (Yiji, Shanghai, China). Briefly, 1 × 10^4^ cells per well were plated in a 96-well plate and cultured at 37 °C overnight. Then, DOX was added to each well. After 24 h incubation, 10 µL of CCK-8 was added to each well for an additional 2 h. Finally, the absorbance (OD) at 450 nm wavelength was measured with a microplate reader (Molecular Devices, USA). The concentration of DOX producing 50% growth inhibition of cells was the IC50 value.

### Real-time quantitative polymerase chain reaction (RT-qPCR)

Real-time quantitative Polymerase chain reaction (RT-qPCR) was used to measure the mRNA expression level of USP10. Firstly, total RNA was extracted by using TRIZOL reagent (Invitrogen, USA), and after reverse transcribed to cDNA, RT-qPCR was conducted with the ABI system. The primer sequence of USP10 forward primer: 5’-GAGGGCACAGC TACCAACG-3’ and reverse primer: 5’-AGGGGAGATAT GGCGGGAG-3’. β-actin forward primer: 5’-TGCTGTCCCTGTATGCCTCT-3’, and reverse primer: 5’-TTTGATGTCACGCACGATTT-3’. Gene expressions were determined using the 2^−∆∆CT^ formula and normalized to β-actin.

### Western blotting

Western blotting was employed to detect the protein expression level [43, 44]. Generally, the total protein of cells was extracted by using protein extraction kit (Pierce, USA), after determining the total protein concentration by using BCA assay kit, equal amounts of proteins were separated by 10% SDS-PAGE and transferred to PVDF membranes, then 1:1000 dilution antibodies were used as follows: anti-USP10, anti-PTEN, anti-p-PI3K, anti-p-AKT, anti-ABCG2, anti-E-cadherin, anti-N-cadherin, anti-Vimentin, anti-p53, anti-caspase-1, anti-Bax, anti-Bcl-xl and anti-β-actin. After using the 1:1000 secondary antibodies, the signals of proteins were measured by using an enhanced chemiluminescence kit and analyzed by using Image J analysis software.

### Transwell assay

Transwell assay was implemented to test the invasion ability of FTC133-DOX. After the top chamber was pre-coated with Matrigel, FTC133-DOX cells were seeded into the top chamber with serum-free medium, and the lower chamber was filled with fetal bovine serum. After culture for 24 h, FTC133-DOX cells invaded the lower chamber were fixed, and then stained with purple crystal. Subsequently, invasive FTC133-DOX cells were photographed and counted by using Image J analysis software.

### Wound healing assay

A wound-healing assay was carried out to determine the migration ability of FTC133-DOX cells. Firstly, FTC133-DOX cells at 1 × 10^6^ cells per well were seeded into 6-well plates. After the confluent, a wound was scrapped by using a micropipette tip. Then, the cell debris was washed using PBS. Then wound morphology was photographed at 0 h and again after 24 h.

### Flow cytometry

Flow cytometry was applied to measure the apoptosis rate of FTC133-DOX cells. Annexin V-fluorescein isothiocyanate/PI apoptosis detection kit (BD Biosciences, USA) was used to stain the apoptotic cells according to the manufacturer’s instructions, and flow cytometry was performed to detect the apoptosis rate.

### Statistical analysis

All data were expressed as mean ± SD and statistical analysis was performed by using GraphPad Prism. Student’s t-test and one-way ANOVA were used for comparison between different groups. A *P*-value less than 0.05 was considered statistically significant.

## Results

### Construction of drug-resistant cell lines and expression of USP10

**Fig. 1:**
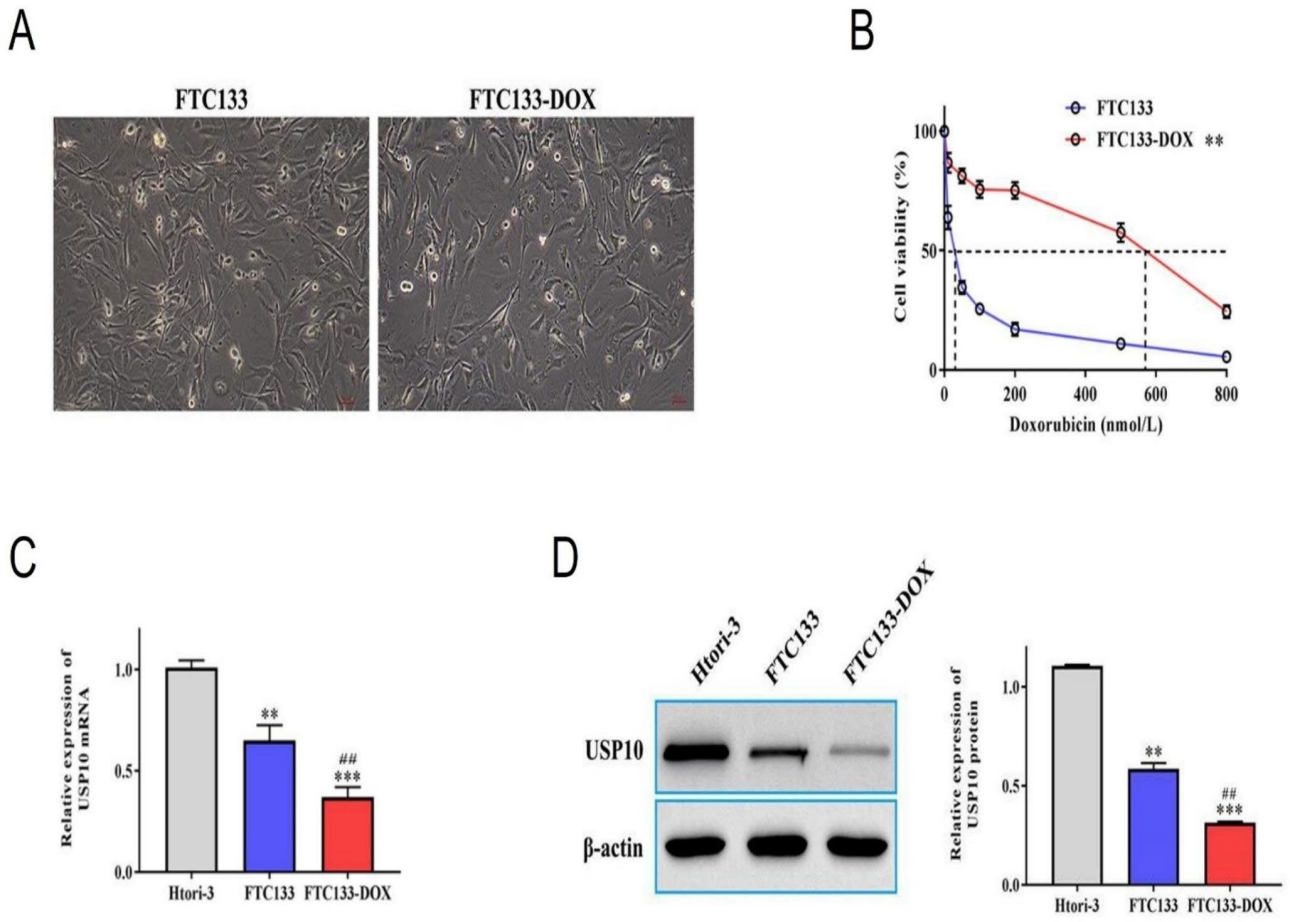
Construction of Doxorubicin resistant FTC133-DOX cells and expression of USP10: (**A**) Morphology of FTC133 and FTC133-DOX cells (Scale bar 50 μm). (**B**) Chemo-sensitivity of FTC133 and FTC133-DOX cells to DOX treatment. ^*^ Compared to FTC133. (**C**) mRNA expression of USP10 in Htori-3, FTC133 and FTC133-DOX cells. ^**^ Compared to Htori-3. ^##^ Compared to FTC133. (**D**) the protein expression of USP10 in Htori-3, FTC133 and FTC133-DOX. ^**^ Compared to Htori-3. ^##^ Compared to FTC133. **P < 0.01; ***P < 0.001 and ^##^*P* < 0.01

In order to study the mechanism of DOX resistance in thyroid cancer cells, we constructed thyroid cancer cell resistant strains (TPC-1-DOX and FTC133-DOX). Microscopic observation of drug-resistant and non-drug-resistant strains showed that the number of cells in drug-resistant strains was higher than that in non-drug-resistant strains, but there was no significant difference in cell morphology (Fig. 1A and Figure S1A). Thyroid cancer cells were then treated with different concentrations of DOX (0,50,100,200,400,800), and it was found that both FTC133 and TPC-1 produced cell resistance, among which FTC133 was more resistant (Fig. 1B and Figure S1B). At the same time, the cell viability of TPC-1 and FTC133 cells decreased with the increase of DOX in a dose-dependent manner. The IC50 of FCT133 to DOX was 28.7 nmol/L, while FTC133-DOX showed higher resistance to DOX, and the IC50 was significantly increased to 489.2 nmol/L, as shown in Fig. 1B. RT-qPCR and western blotting results showed that the expression of USP10 in FTC133, FTC133-DOX, TPC-1 and TPC-1-DOX was lower than that in human normal thyroid cell line Htori-3, and the expression of USP10 in FTC133-DOX was lower than that in FTC133 ( Fig. 1C-D ). Similarly, we also found that the expression of USP10 in TPC-1-DOX was lower than that in TPC-1 (Figure S1C-D). Notably, these effects were more prominent in FTC133 cell line compared to the TPC-1 cells, therefore, we selected the FTC133 cell line for subsequent experiments.

### USP10 affected the sensitivity of thyroid cancer cells to DOX

**Fig. 2:**
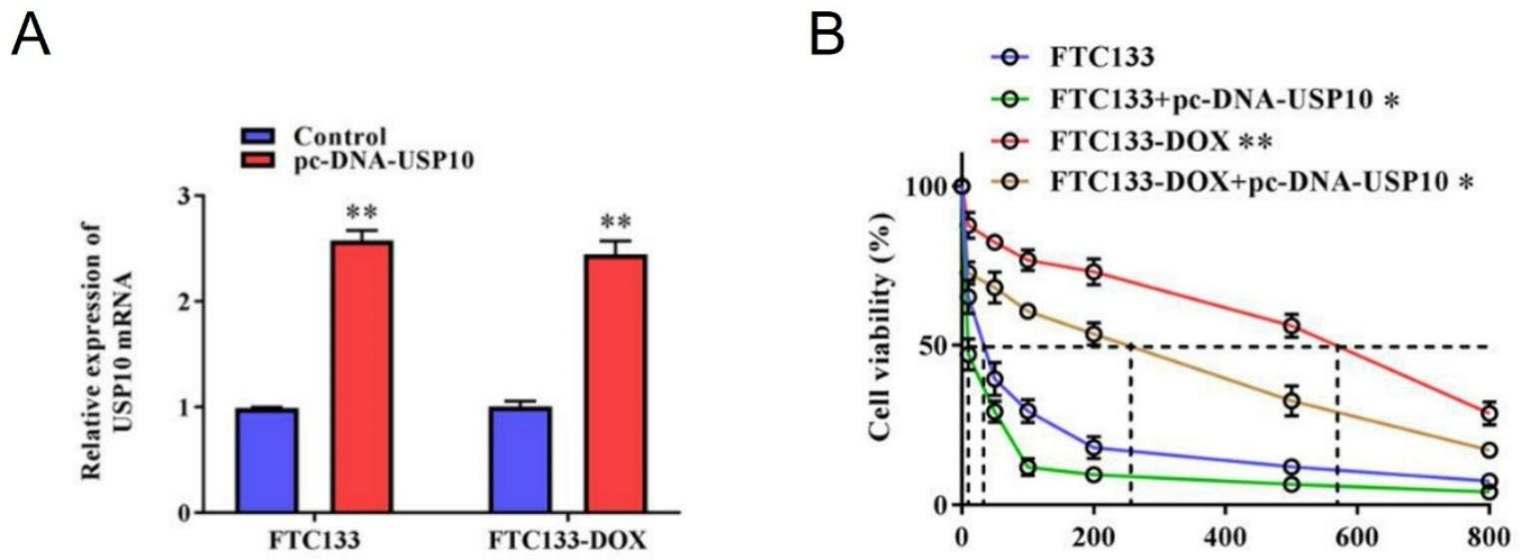
Overexpression of USP10 affects the sensitivity of FTC133-DOX cells to DOX. (**A**) Transfection efficiency of pc-DNA-USP10. * Compared to the empty vector control group. (**B**) Chemo-sensitivity of FTC133 and FTC133-DOX cells to DOX that were transfected with or without pc-DNA-USP10. * Compared to FTC133. # compared to FTC133-DOX. *P < 0.05, and **P < 0.01

In order to elevate the role of USP10 in sensitivity to DOX of thyroid cancer cells and based on the experimental results of its abnormally low expression, pcDNA-USP10 was transfected into FTC133 and FTC133-DOX cells to overexpress expression of USP10. RT-qPCR assay showed that the expression of USP10 was upregulated after successfully transfecting the cells (Fig. 2A). After successfully overexpressing USP10, we then evaluated the effect of USP10 on chemo-sensitivity to DOX. Compared with FTC133 and FTC133-DOX cells, the IC50 of cells transfected with the pcDNA-USP10 was significantly reduced to 13.7 nmol/L and 201.4 nmol/L respectively, as shown in Fig. 2B.

### USP10 regulated PTEN/PI3K/AKT/ABCG2 signaling axis in FTC133-DOX cells

**Fig. 3:**
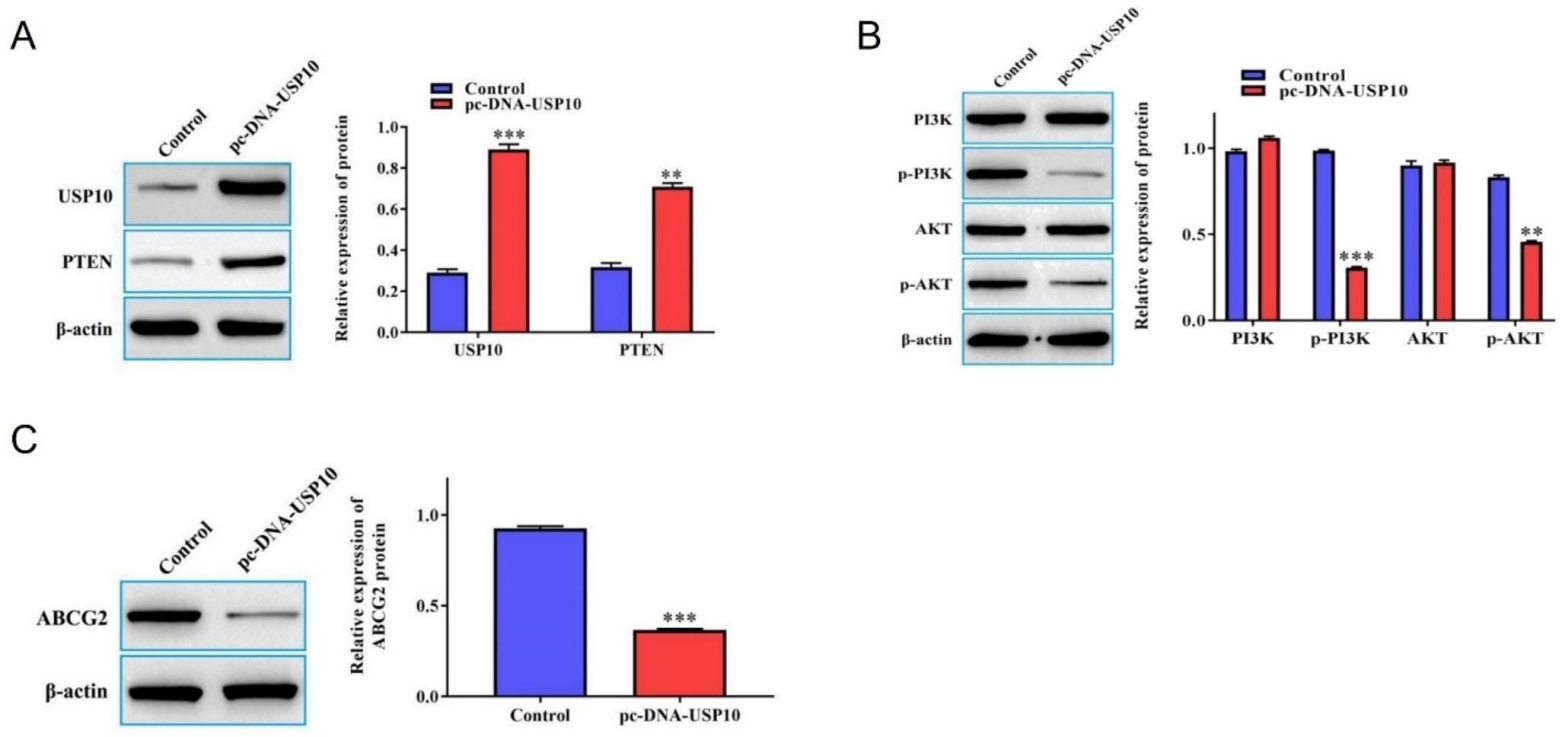
USP10 regulates PTEN/PI3K/AKT/ABCG2 signaling pathway in FTC133-DOX cells. (**A**) Western blot was used to detect the protein expression level of PTEN in FTC133-DOX cells that were transfected with either empty vector or with pc-DNA-USP10. (**B**) The protein expression level of PI3K, p-PI3K, AKT, and p-AKT in FTC133-DOX cells that were transfected with either empty vector or with pc-DNA-USP10. (**C**) The protein expression level of ABCG2 in FTC133-DOX cells that were transfected with either empty vector or with pc-DNA-USP10. * Compared to the Control group. **P < 0.01; and ***P < 0.001

To study the underlying mechanism by which USP10 could affect the biological characteristics of DOX-resistant FTC133-DOX thyroid cancer cells, we investigated PTEN/PI3K/AKT/ABCG2 signaling axis. Results demonstrated that there was reduced expression of PTEN in FTC133-DOX cells which was significantly upregulated in the pcDNA-USP10 transfected cells (Fig. 3A). Similarly, the protein expression levels of p-PI3K and p-AKT were high in FTC133-DOX cells, which were prominently reduced by overexpressing USP10 in FTC133-DOX cells (Fig. 3B). Moreover, the protein expression level of ABCG2 which was high in FTC133-DOX cells was found to be downregulated by pcDNA-USP10 in FTC133-DOX cells (Fig. 3C). The proteins expression of USP10 and PTNE in FTC133-DOX that transfected with or without pc-DNA-USP10.

### USP10 affected the biological properties of FTC133-DOX cells via regulating ABCG2

**Fig. 4:**
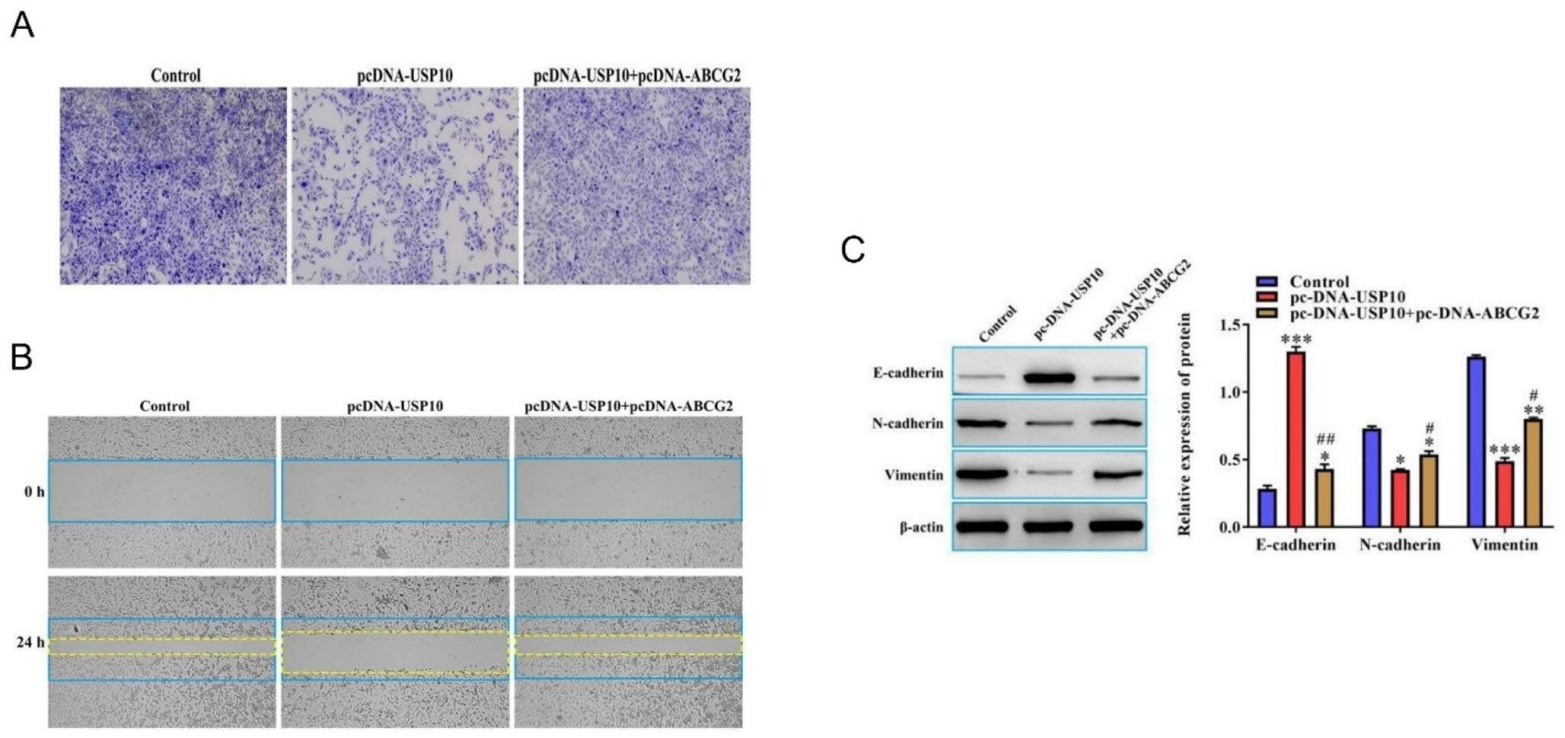
USP10 affects the invasion, migration, and EMT properties of FTC133-DOX cells via regulating ABCG2. (**A**) Invasive capacity of FTC133-DOX cells that were transfected with or without pc-DNA-USP10 or pc-DNA-ABCG2 (×40). (**B**) Migration capacity of FTC133-DOX cells that were transfected with or without pc-DNA-USP10 or pc-DNA-ABCG2 (×40). (**C**) Western blot assay was used to detect EMT-related proteins such as E-cadherin, N-cadherin, and Vimentin in FTC133-DOX cells that were transfected with or without pc-DNA-USP10 or pc-DNA-ABCG2 * Compared to the Control group. # compared to pc-DNA-USP10 group. *P < 0.05; **P < 0.01; ***P < 0.001; ^#^*P* < 0.05; and ^##^*P* < 0.01

We next evaluated the effect of USP10 on the invasive, migration, and epithelial-mesenchymal transition (EMT) biological properties of FTC133-DOX cells. For this purpose, Transwell and wound healing assays were employed to elevate the invasion and migration ability of FTC133-DOX cells. We found that the number of invasive cells and migration distance was lower in the pcDNA-USP10 group compared to the control group, while these properties were higher in the pcDNA-USP10 + pcDNA-ABCG2 group compared with the pcDNA-USP10 group (Fig. 4A-B). Furthermore, the western blot results showed that the expression level of E-cadherin was upregulated, but N-cadherin and Vimentin were downregulated in the pcDNA-USP10 group compared to the empty vector control group. Notably, these events were reversed by overexpressing ABCG2 as shown in the pc-DNA-USP10 + pc-DNA-ABCG2 group compared to the pc-DNA-USP10 group (Fig. 4C). These results indicate that USP10-mediated biological properties of FTC133-DOX are regulated by ABCG2.

### USP10 induced cell apoptosis in FTC133-DOX via regulating ABCG2

**Fig. 5:**
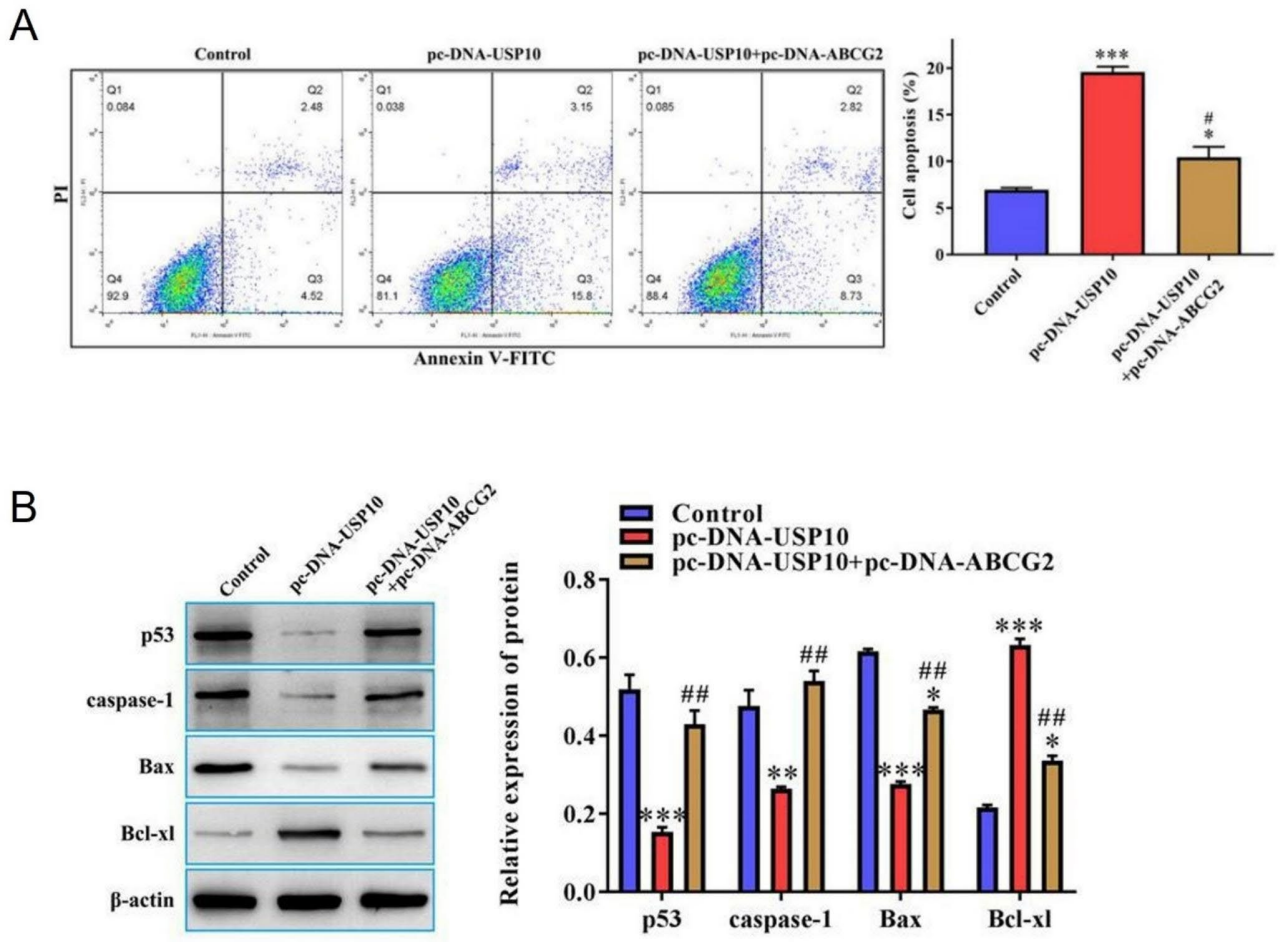
USP10 induces cell apoptosis in FTC133-DOX cells via regulating ABCG2. (**A**) Flow cytometry was used to detect the apoptosis rate in FTC133-DOX cells that were transfected with or without pc-DNA-USP10 or pc-DNA-ABCG2. (**B**) Western blot was used to measure the protein expression levels of p53, caspase-1, Bax, and Bcl-xl in FTC133-DOX cells that were transfected with or without pc-DNA-USP10 or pc-DNA-ABCG2. * Compared to Control group. # Compared to pc-DNA-USP10 group. *P < 0.05; **P < 0.01; ***P < 0.001; ^#^*P* < 0.05; and ^##^*P* < 0.01

Finally, we explored the role of USP10 and ABCG2 in inducing apoptosis in FTC133-DOX cells. Flow cytometry results showed that pcDNA-USP10 induced apoptosis in FTC133-DOX cells compared to the empty vector control group (Fig. 5A). Interestingly, this elevated apoptosis rate of FTC133-DOX was reduced after co-transfecting pc-DNA-USP10 cells with pc-DNA-ABCG2. Similarly, western blotting results indicated that the expression of pro-apoptotic proteins p53, caspase-1, and Bax decreased, and anti-apoptotic protein Bcl-xl increased after overexpressing USP10 compared to the control group (Fig. 5B). However, after co-transfecting pc-DNA-USP10 cells with pc-DNA-ABCG2, the expression of pro-apoptotic proteins elevated while anti-apoptotic protein was reduced by overexpressing ABCG2 at the same time (Fig. 5B), suggesting that USP10 induced apoptosis in FTC133-DOX cells is mediated by ABCG2.

## Discussion

Thyroid cancer which manifests surprisingly malignant biological behavior directly responsible for most thyroid cancer-related deaths (Chmielik et al. 2018). Developing drug resistance adds difficulty to the treatment of thyroid cancer (Ancker et al. 2019). Extensive studies indicated that USP10, as a deubiquitinating enzyme, is associated with the growth of tumor cells and play an important role in various cancers. For example, Zeng et al., suggested that USP10 was an independent predictor of the prognosis of GC patients (Zeng et al. 2014), besides, loss of USP10 protein expression was an independent prognostic biomarker for poor prognosis in patients with epithelial ovarian cancer (Han et al. 2019). Focus on drug resistance of cancer, there were also several studies that demonstrated that USP10 participated in cisplatin resistance of osteosarcoma cells, colon cancer cells, cervical cancer cells, and non-small cell lung cancer cells (Annibaldi et al. 2011; Hu et al. 2020). But the research on USP10 in thyroid cancer is very rare. In this study, we found that the expression of USP10 was lower in thyroid cancer cells than that in normal thyroid cells, and it was lower in DOX resistance thyroid cancer cells than that in parental thyroid cancer cells. Further, we measured the chemosensitizing potential of USP10 in FTC133 and FTC133-DOX, and we found that overexpression of USP10 increased their sensitivity to DOX. This strongly suggests that USP10 plays a critical role in DOX resistance to the thyroid cancer cell.

Previously, it has been reported that USP10 loss was linked to lymphovascular invasion and distant metastases in patients with colorectal cancer (Kim et al. 2020). Besides, USP10 has been shown to be involved in cancer cell migration by regulating the stability of the EMT-transcription factor Slug/SNAI2 and Vimentin (Ouchida et al. 2018). In this study, we also found that overexpression of USP10 inhibited invasion, migration, and EMT properties of DOX-resistant thyroid cancer cells. In addition, USP10 has been shown to regulate p53 localization and stability by deubiquitinating p53 (Yuan et al. 2010). Previously, it has been demonstrated that up-regulation of USP10 expression, results in the activation of the p53 pathway, contributing to the inhibition of thyroid cancer growth (Cui et al. 2014). In line with this study, here, we also found that overexpression of USP10 downregulated p53, caspase-1, and Bax, while upregulated Bcl-xl in DOX-resistant thyroid cancer cells. A previous study has found that modulation of p53 and Bcl-xl expression could induce apoptosis of thyroid cancer cells (Liu et al. 2010). Our results also indicated that overexpression of USP10 promoted apoptosis of DOX resistance thyroid cancer cells. As an ABC transporter, endogenous ABCG2 expression in certain cancers is likely a reflection of the differentiated phenotype of the cell of origin and likely contributes to intrinsic drug resistance (Robey et al. 2009). Importantly, the translocation of the ABCG2 drug transporter away from the plasma membrane resulted in a concomitant decrease in doxorubicin extrusion in thyroid cancer cell lines (Lopez et al. 2007). Our study also showed that overexpression of USP10 inhibited the malignant biological behavior of DOX-resistant thyroid cancer cells, while concomitant overexpression of ABCG2 reversed this inhibition caused by USP10.

PTEN is a dual protein/lipid phosphatase whose main substrate is phosphatidylinositol, a product of PI3K. The increase of 3,4,5 triphosphate (PIP3) recruits AKT to the membrane and is activated by other PIP3-dependent kinases on the membrane (Carnero et al. 2008). The PI3K/AKT pathway is an important signaling pathway for drug resistance in various cancers such as melanoma, lung cancer, ovarian cancer, leukemia, and hepatocellular carcinoma (Chen et al. 2018a, b; Rittler et al. 2019; Soltani et al. 2019). Excessive activation of PI3K/AKT is an important factor in cell resistance in cancer (Liu et al. 2020). This study found that USP10 affects the drug resistance of thyroid cancer cells by regulating the PTEN/PI3K/AKT pathway. In addition, several studies have shown that the PI3K/AKT pathway enhances the biological basis of cancer through ABCG2, and its activation may reduce the response to chemotherapy drugs and enhance drug efflux (Lampada et al. 2017; Tazzari et al. 2007). Our results showed that overexpression of USP10 up-regulated the expression of PTEN and down-regulated the expression of p-PI3K, p-AKT and ABCG2. At the same time, our study also showed that overexpression of USP10 inhibited the malignant biological behavior of DOX-resistant thyroid cancer cells by inhibiting the expression of ABCG2 by regulating the PTEN/PI3K/AKT pathway.

The ABCG2 transporter is specifically distributed on the plasma membrane and is considered to be one of the most important transporters leading to cell resistance (Choudhuri and Klaassen 2006; Fetsch et al. 2006). As an ABC transporter, endogenous ABCG2 expression in certain cancers is likely a reflection of the differentiated phenotype of the cell of origin and likely contributes to intrinsic drug resistance (Robey et al. 2009). Studies have shown that ABCG2 can transport a variety of anti-tumor drugs, such as epirubicin, daunorubicin, irinotecan, doxorubicin, topotecan, 9-aminocamptothecin and mitoxantrone (Doyle and Ross 2003; Zeng et al. 2020). In the process of mediating drug resistance of tumor cells, ABCG2 recognizes and effectively squeezes out various chemotherapeutic drugs with different chemical structures, resulting in drug resistance of cancer cells (Zeng et al. 2020). Importantly, the translocation of the ABCG2 drug transporter away from the plasma membrane resulted in a concomitant decrease in doxorubicin extrusion in thyroid cancer cell lines (Lopez et al. 2007). This study found that overexpression of USP10 can inhibit the expression of ABCG2. At the same time, our study also showed that overexpression of USP10 inhibited the malignant biological behavior of DOX-resistant thyroid cancer cells, while concomitant overexpression of ABCG2 reversed this inhibition caused by USP10.

## Conclusions

In our study, we found that the expression of USP10 in DOX-resistant thyroid cancer cells is lower than that in their parental cells. Overexpression of USP10 increases the sensitivity of FTC133 and FTC133-DOX to DOX therapy. Mechanistically, overexpressing USP10 inhibits the invasion, migration and EMT of thyroid cancer cells by regulating the expression of ABCG2 and mediating PTEN/PI3K/AKT signaling pathway. Moreover, overexpression of USP10 promotes the apoptosis of DOX-resistant thyroid cancer cells.

### Electronic supplementary material

Below is the link to the electronic supplementary material.

Supplementary Material 1

## Data Availability

The datasets generated during and/or analyzed during the current study are available from the corresponding author upon reasonable request.
