# Supplementary material for: USP10 suppresses ABCG2-induced malignant characteristics of doxorubicin-resistant thyroid cancer by inhibiting PI3K/AKT pathway
Source: J Bioenerg Biomembr. 2023 Nov 3;55(6):457–66. doi: 10.1007/s10863-023-09986-3 (PMC10682060; doi:10.1007/s10863-023-09986-3)
Supplement: Supplementary file 1 — Supplementary Material 1 [file 10863_2023_9986_MOESM1_ESM.docx]

**Figure S1.**


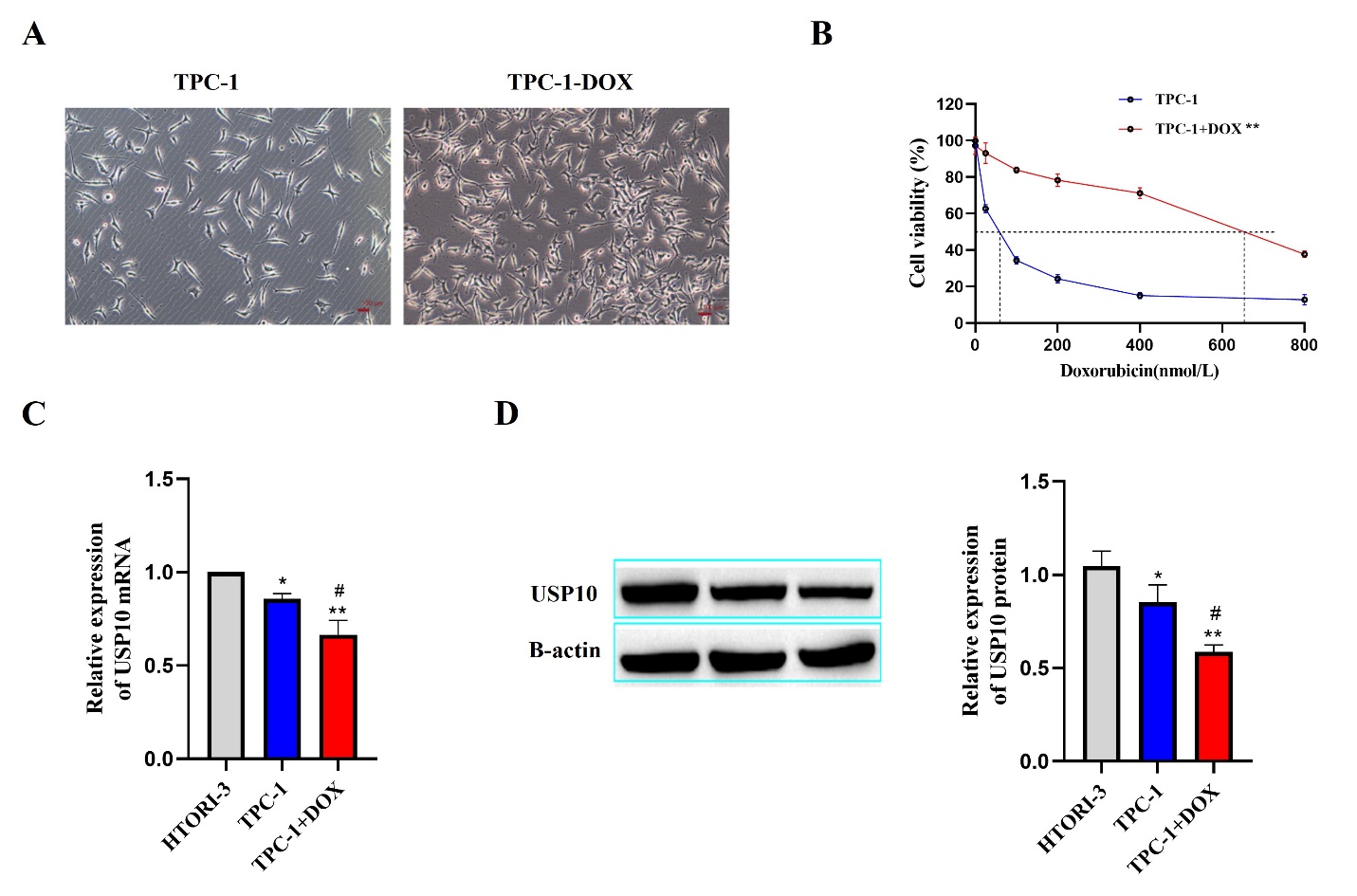


Figure S1. Construction of Doxorubicin resistant TPC-1-DOX cells and expression of USP10. (A) Morphology of TPC-1 and TPC-1+DOX cells (Scale bar 50 μm). (B) Chemo-sensitivity of TPC-1 and TPC-1+DOX cells to DOX treatment. * Compared to TPC-1. (C) mRNA expression of USP10 in Htori-3, TPC-1, and TPC-1+DOX cells. * Compared to Htori-3. # Compared to TPC-1. (D) The protein expression of USP10 in Htori-3, TPC-1, and TPC-1+DOX cells. * Compared to Htori-3. # Compared to TPC-1. *P<0.05; **P<0.01; and ^#^*P*<0.05.
